# International Health Regulations (2005) and the U.S. Department of Defense: building core capacities on a foundation of partnership and trust

**DOI:** 10.1186/1471-2458-10-S1-S4

**Published:** 2010-12-03

**Authors:** Matthew C Johns, David L Blazes

**Affiliations:** 1 Armed Forces Health Surveillance Center, Division of Global Emerging Infections urveillance and Response System. 2900 Linden Lane, Silver Spring, MD 20910, USA

## Abstract

A cornerstone of effective global health surveillance programs is the ability to build systems that identify, track and respond to public health threats in a timely manner. These functions are often difficult and require international cooperation given the rapidity with which diseases cross national borders and spread throughout the global community as a result of travel and migration by both humans and animals. As part of the U.S. Armed Forces Health Surveillance Center (AFHSC), the Department of Defense’s (DoD) Globa Emerging Infections Surveillance and Response System (AFHSC-GEIS) has developed a global network of surveillance sites over the past decade that engages in a wide spectrum of support activities in collaboration with host country partners. Many of these activities are in direct support of International Health Regulations (IHR[2005]). The network also supports host country military forces around the world, which are equally affected by these threats and are often in a unique position to respond in areas of conflict or during complex emergencies. With IHR(2005) as the guiding framework for action, the AFHSC-GEIS network of international partners and overseas research laboratories continues to develop into a far-reaching system for identifying, analyzing and responding to emerging disease threats.

## Introduction and background

The central focus of disease surveillance systems is the early identification of infectious disease outbreaks in order to rapidly implement effective control measures for minimizing disease transmission and morbidity. Today, a heightened challenge in understanding these disease dynamics is the interconnected nature of our global society. Diseases cross international borders and present in unique ways through a continuously changing landscape. This challenge makes it difficult to rapidly identify, analyze and respond to disease outbreaks. Appropriate and effective monitoring of newly recognized disease events requires established, standardized and well-maintained global surveillance systems with flexible components for identifying and responding to such events under the guiding principles of the World Health Organization’s (WHO) International Health Regulations (IHR[2005]) [1].

**Figure 1:**
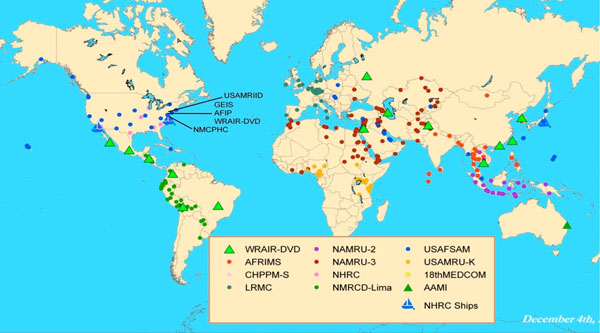
AFHSC-GEIS Global Network of Partners: Global Influenza Surveillance Sites.

In 1997, the U.S. Department of Defense (DoD) established the Global Emerging Infections Surveillance and Response System (GEIS) in response to the U.S. Presidential Decision Directive NSTC-7, which detailed the need for more robust global disease surveillance [2]. In 2008, GEIS, as a global system, was integrated into the newly formed Armed Forces Health Surveillance Center (AFHSC) [3]. The primary mission of AFHSC-GEIS is global disease surveillance and assessment of public health events affecting U.S. service members and host country partners around the world. A large portion of this mission is accomplished through the DoD overseas research laboratories, which were initially established within partner host countries to conduct research on infectious diseases of local and regional concern [4]. This capacity has subsequently been leveraged by AFHSC-GEIS for the purpose of disease surveillance and response through a growing network of centrally funded partners (Figure 1).

There are currently five DoD overseas research laboratories that serve as primary AFHSC-GEIS partners: the Armed Forces Research Institute of Medical Sciences (AFRIMS) in Bangkok, Thailand; the U.S. Army Medical Research Unit – Kenya (USAMRU-K) in Nairobi, Kenya; the U.S. Naval Medical Research Center Detachment (NMRCD) in Lima, Peru; the U.S. Naval Medical Research Unit No. 2 (NAMRU-2) which conducts surveillance in Southeast Asian countries; and the U.S. Naval Medical Research Unit No. 3 (NAMRU-3) in Cairo, Egypt. Additionally, the AFHSC-GEIS network includes substantial contributions from three U.S.-based research laboratories and a major regional medical center in Europe. The Naval Health Research Center (NHRC) in San Diego, California conducts population-based surveillance among basic military trainees at eight of the ten major military training centers in the U.S., disease surveillance among shipboard service members in the U.S. naval fleets, and infectious disease surveillance in six clinics and two hospitals along the U.S.-Mexico border (in collaboration with the U.S. Centers for Disease Control and Prevention [CDC] and the County of San Diego Health Department). The U.S. Air Force School of Aerospace Medicine (USAFSAM) in San Antonio, Texas (formerly the Air Force Institute for Operational Health) serves as the Air Force’s clinical reference laboratory and public health center and is the lead organization for the U.S. military’s installation-based influenza sentinel surveillance program. The Walter Reed Army Institute of Research’s Division of Viral Diseases (WRAIR-DVD) in Silver Spring, Maryland serves as a central DoD hub for providing full-length genomic sequencing capability and conducts surveillance among U.S. civilians assigned to Department of State embassies, consulates and missions overseas. Additionally, a partnership between the Landstuhl Regional Medical Center (LRMC) and the U.S. Army Public Health Command Region-Europe (PHRC-Europe) in Landstuhl, Germany function as a regional military medical center and support surveillance for respiratory pathogens and other emerging infectious diseases (EID) within the U.S. European Command, or EUCOM.

Building the core capacities at the local level as described in Annex 1 of the IHR(2005) is a primary focus and strategic goal of the AFHSC-GEIS network [5]. Support for these efforts develops the capability for open lines of communication between IHR States Parties, but takes years of system strengthening and development. The interaction and communication between States Parties, as defined in Article 44 in the IHR(2005), allow for collaborative exchanges and system strengthening, and most importantly, build bilateral relationships on a firm foundation of trust at the local level. We feel these are critical components of the long-standing relationships between the AFHSC-GEIS network partners and their sponsor host countries and all move the greater global health community closer to implementation of the IHR(2005).

## System components

### Surveillance

#### Laboratory-based surveillance

Beginning in 2006, the DoD’s global disease surveillance network has worked to enhance the existing surveillance infrastructure to prepare for a potential influenza pandemic. The goals of these expansion efforts include broadening the network to monitor and detect increasing numbers of avian (H5N1) influenza outbreaks around the world and identifying new infectious disease threats [6]. This expansion of capacity and function was both appropriate and fortuitous as AFHSC-GEIS network partners at NHRC and USAFSAM were the first in the world to identify the novel influenza A/H1N1 strain in April 2009 in California and Texas [7,8]. This rapid detection and subsequent response during the tail end of the influenza season allowed the appearance of this novel strain to be identified and reported as a potential public health emergency of international concern (PHEIC) by the U.S. IHR National Focal Point to the WHO. With the onset of this influenza A/H1N1 pandemic in April of 2009, there were significant efforts by AFHSC-GEIS network partners to assist the global health community in responding to this global threat.

#### Electronic surveillance

Electronic disease surveillance is also an important component of a comprehensive global public health disease prevention and control strategy, and contributes significantly to capacity building and support for IHR(2005) implementation in partner countries. Using electronic methods for data collection and analysis allows for accurate and timely outbreak detection as well as providing meaningful situational awareness during, or in the aftermath of, an outbreak or pandemic. The AFHSC-GEIS network has supported a number of electronic disease surveillance initiatives over the past several years in partnership with several DoD overseas laboratories, host country health and defense ministries and our technical partner, the Johns Hopkins University Applied Physics Laboratory (JHU/APL) [9].

AFHSC-GEIS has relied on the extensive experience that JHU/APL acquired in the design and implementation of the Electronic Syndromic Surveillance for Early Notification of Community-based Epidemics (also known as ESSENCE) system [10]. This electronic disease surveillance system is used worldwide at all DoD military treatment facilities, throughout the entire U.S. Veterans Health Administration system, and in at least twelve U.S. state health facilities. Tools have been created to enable data collection from the most sophisticated sources down to remote settings where data have traditionally been difficult, if not impossible, to collect and analyze. These tools have broad-reaching applicability in any resource-limited setting, whether it is overseas or in response to a complex emergency in the United States [11]. The following describes some of the efforts that have focused on adapting electronic surveillance techniques to resource-limited settings.

Two electronic surveillance efforts were developed by partners in Southeast Asia and optimized in 2009, including a project with the Royal Thai Army (RTA) in remote border areas as well as a pilot Short Message Service (SMS)-based project in the Philippines. This project was developed by the RTA with support from AFRIMS and AFHSC-GEIS, and reports diseases in both military and local civilian populations through faxing reports or through voice via military radio. The next generation of the system was fielded recently, and simplified data collection from 216 symptoms and categorization into 12 syndromes that are consistent with the Thai Ministry of Health’s reporting requirements. This updated system also added questions about poultry exposure, leptospirosis, novel A/H1N1 infection, and chickungunya virus infection. Although no major outbreaks of disease were detected by this system in 2009, it continued to provide situational awareness for the RTA and Thai Ministry of Health [9].

### Infrastructure development

Capacity building initiatives continue to be a major focus area for the AFHSC-GEIS contributions to worldwide EID surveillance and response activities. Surveillance, response, laboratory capacity and human resource development are listed as four of the main core capacities within the IHR(2005). With regards to laboratory capacity, it has been suggested in developing countries this function may be the “Achilles’ Heel” of global efforts to combat infectious diseases [12]. As a result of this, many of the AFHSC-GEIS sponsored activities in capacity building have been directed at improving the existing infrastructure through renovation of existing laboratory facilities, furnishing of new scientific equipment, and provision of new or enhanced diagnostic testing systems for overseas host country laboratories. In 2009 alone, efforts were coordinated with over 80 local and regional health, agriculture and defense ministries, as well as other governmental officials and institutions in 74 countries worldwide. During the same time period, a total of 52 National Influenza Centers (NICs) and other country-specific influenza and other EID reference laboratories (44 civilian, 8 military) were supported in 46 countries [9]. Partner countries in all major regions of the world were supported but special emphasis was placed on capacity building efforts in Sub-Saharan (East, Central and West) Africa, with activities taking place in fourteen different countries in-line with stated global health priorities and areas of enhanced focus for such efforts [13,14].

**Figure 2:**
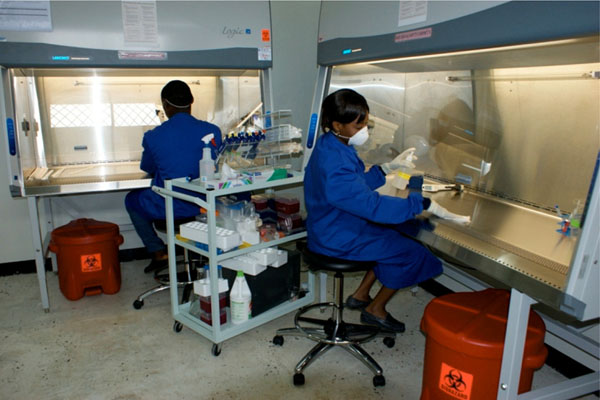
Newly Commissioned Biosafety Level-2 Laboratory at the University of Buea, Cameroon.

This past year, AFHSC-GEIS established two new biosafety level 3 (BSL-3) laboratory suites within DoD partner countries. The first suite was established by the AFRIMS in Bangkok, Thailand and was U.S.-certified and commissioned on July 8, 2009. The suite was officially inaugurated in September of 2009 and began immediately supporting work in avian and pandemic influenza monitoring, including culture and molecular sequencing capability. This BSL-3 laboratory constitutes the first certified laboratory of its kind in the region and provides the host country and other countries in Southeast Asia with a much-needed high containment capability to conduct research and assist with outbreaks involving select human and animal bacterial and viral strains. A second BSL-3 laboratory suite was opened in late 2009 in San Diego, California by partners at NHRC, allowing work with zoonotic influenza strains submitted by AFHSC-GEIS partners throughout the world, to include development of new virus testing capabilities against H5N1 and other highly pathogenic avian influenza strains. Additionally, two BSL-2 laboratories were established in Cameroon, including the Cameroon Army Military Health Research Center in Yaoundé and at the University of Buea in Western Cameroon (Figure 2). Both laboratories will help to improve the ability to conduct influenza and EID diagnostic work in the country [5].

Developing and deploying influenza diagnostic capabilities through other NICs were also supported by the NAMRU-3 in Afghanistan, Jordan, and Iraq; by the U.S. NMRCD-Peru in Venezuela, Colombia, Paraguay, and Ecuador; and, in Kenya by the USAMRU-Kenya. Finally, in collaboration with the CDC’s Central America and Panama Center, the U.S. Army Public Health Command Region–South (PHCR-South) provided laboratory technical assistance, reagents and supplies to the health ministries in El Salvador, Guatemala, Honduras, Nicaragua, and Panama, resulting in the certification of the Guatemalan NIC and in the testing of over 5,000 specimens for novel A/H1N1 [5].

### Outbreak support

Rapid identification of outbreaks and support of timely response efforts are key components of complying with IHR(2005), and are core focus areas of the AFHSC-GEIS network [5,15]. These efforts are provided in response to host country requests for assistance with new or ongoing outbreaks and consists of a wide range of functional support. These support efforts include field team support, epidemiology or consultative support and/or laboratory diagnostic support. These collaborative exchanges strengthen relationships, build and maintain trust and are a critical component of the long-standing relationships built between the network partners and their sponsor host countries. Several of the partnerships between the U.S. and host country militaries (mil-mil partnerships) that have developed over many years have served to empower the host country military’s role in supporting outbreak response activities within their own countries and ultimately support the guiding principles of surveillance and response core capacities within the IHR(2005) [16].

Over the past full year of surveillance (September 2008-October 2009), the AFHSC-GEIS network responded to 76 outbreaks in 53 countries, several in direct support of the IHR(2005). The most common events investigated were influenza (47), cholera (4), dengue fever (4) and hepatitis (3). Human disease was detected in all but one of these outbreaks and specific causative agents were identified in 92%, or 69, of them. The population size affected ranged from less than 10 individuals to several thousand and support efforts were often ongoing engagements beyond the initial phase of the investigation [15].

Beginning in April of 2009, with the onset of the influenza pandemic, disease surveillance and investigative response activities were dominated by novel influenza A/ H1N1. The AFHSC-GEIS network supported the diagnostic confirmation (directly in DoD laboratories or through support of host country laboratories) of the first influenza A/H1N1 cases in fourteen countries (United States, Bhutan, Cambodia, Djibouti, Kuwait, Kenya, Lao People’s Democratic Republic, Lebanon, Egypt, Nepal, Colombia, Ecuador, Peru and the Republic of the Seychelles), again demonstrating direct support for increasing compliance with IHR(2005) for what was deemed at the time as a PHEIC. The non-U.S. activities were a result of the respective AFHSC-GEIS partner laboratory’s role as the regional reference testing center and the bilateral working collaborations with host country health ministries and partner governments. These bilateral relationships resulted in response to seventeen large-scale outbreaks among civilians in thirteen countries [15].

United States service members and beneficiaries were affected by novel H1N1 in all regions of the world within the first month of the outbreak. In the first wave of the pandemic (April through August 2009), the AFHSC-GEIS network partners actively investigated eighteen different outbreaks on U.S. military installations and among defined high-risk groups [17]. These high-risk groups were defined as deployed or deploying personnel, shipboard personnel, new accessions (basic and advanced military trainees and service academy students), health care workers, children and staff in daycare centers and pregnant women. Stressful military environments, highly-mobile missions and complex troop dynamics have proven in the past to help to propagate pandemics and have drawn the attention of the military’s operational leadership and leaders of civilian sectors within host countries [18]. These investigations involved anywhere from a few dozen cases to situations where greater than one thousand cases were tested at a given time.

### Training

In order to achieve optimal and coordinated implementation of the IHR(2005), it is required that each State Party be capable of detecting, confirming, reporting, and containing an emerging threat to public health [1] and build these capabilities by 2012. To reach the 2012 milestones, States Parties will need to significantly enhance their laboratory infrastructure and more importantly, appropriately train the personnel who can perform the core capacity functions as defined by Article 5 of IHR(2005). These core capacities include leading outbreak investigations, correctly identifying a pathogen in the laboratory, rapidly communicating the findings to stakeholders at all levels and, most importantly, controlling the outbreak through tested, coordinated and exercised mitigation efforts. Frontline public health professionals require the latest knowledge and skills to address global threats to public health.

**Figure 3:**
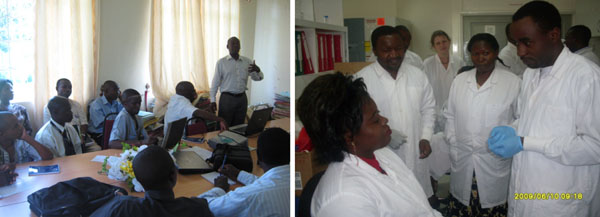
Two week Makarere University, Uganda training for thirty six laboratory scientists from throughout Sub-Saharan Africa.

The recent pandemic of novel influenza A/H1N1 clearly illustrates the unpredictable nature of pathogens which require dynamic and evolving public health strategies for surveillance, disease management, and effective mitigation. Training public health professionals to understand, monitor, respond to, control, and prevent emerging infections is a foundational goal of AFHSC-GEIS [19]. Since its inception, AFHSC-GEIS partners and collaborators have made available their overseas laboratory and field study facilities to serve as regional focal points for public health training of staff , technicians and epidemiologists within partner host countries [2,6,20] through a growing collaborative network of U.S. Government agency partners (Figure 3). A wide range of training has been coordinated to include programs such as the CDC’s Field Epidemiology Training and Laboratory Training Programs [21] and the U.S. Agency for International Development (USAID) efforts throughout Africa and the Pacific [22-24]. These opportunities through training serve as a forum for support, coordination, and collaboration with host country partners as prescribed in Article 44 of the IHR(2005).

A regional approach to training has been underway by the Uniformed Services University of the Health Sciences (USUHS) Center for Disaster and Humanitarian Assistance Medicine (CDHAM). Educational efforts in support of five combatant commands have been bolstered with AFHSC-GEIS funding and provide much-needed professional expertise and the latest technical information to U.S. military and civilian health care providers as well as to host country of health, agriculture, defense ministries and other civilian agency collaborators. These initiatives result in professional engagement for host country officials as well as further enhancing the U.S. government’s role as a key stakeholder in the global health community. Training initiatives are broadly based to en-compass such topics as planning and preparedness, outbreak investigation, surveillance and response and include curriculum for a wide spectrum of disease or syndrome causing agents [19].

Over the past full year of partner funding, 18 different organizations conducted 123 training initiatives in 40 countries reaching approximately 3,130 U.S. military and civilian personnel as well as host country personnel. The most common categories covered were EID laboratory techniques (41%), pandemic influenza (24%), and disease surveillance techniques (19%). Training tools included workshops (defined here as hands-on, interactive training), academic courses, conferences, tabletop exercises, and distance learning. Training duration ranged from 0.5 hours to 6 months, with the majority of education efforts lasting 2 to 5 days. Respiratory infections (namely influenza) represented the majority of training initiatives (68%), followed by febrile and vector-borne infections (11%), gastrointestinal infections (5%), and antimicrobial resistance (2%) with an additional 14% represented by various other topics (Geographic Information Systems, EpiInfo, Field Epidemiology, Principles and Practice of Clinical Research, Outbreak Investigation, Emerging Infectious Diseases, Lab Safety Precautions, Ecological Niche Modeling, and Tropical Medicine Student Rotations). Training initiatives occurred in each of the six regional Combatant Commands with most occurring in the Pacific Command, or PACOM [19].

### Communication tools

Communication and central coordination remains a primary role of AFHSC and involves a substantial amount of effort from all divisions and areas, including GEIS. In a given month during the Northern Hemisphere respiratory season, the AFHSC creates, on average, 139 individual reports for a wide spectrum of disease-causing agents or matters of public health concern. This includes routine reportable medical event reports that are noted in the Medical Surveillance Monthly Reports [25]. Many of the reports generated by AFHSC are available on the program website and are open to access by the general public. Other reports are provided to the individual services and are usually part of specific data request agreement with that service.

Respiratory surveillance, and more specifically influenza surveillance, remains the primary area of funding and focus for many of the global network partners. The central coordination and analysis of aggregated influenza surveillance data and summarizing of all partner reports is a function unique to AFHSC central headquarters. AFHSC-GEIS partners are highly encouraged to create program-specific reports that are accurate, timely, concise, openly available, and provide meaningful public health interpretation of the findings. AFHSC-GEIS staff analyze and summarize all partner reports weekly and provide a DoD-wide summary to stakeholders across the network through the AFHSC website [26]. In addition to timely reporting, partners are required to participate and communicate their recent findings through network calls and a bi-monthly teleconference called Epi Chiefs. This provides a forum for reporting the latest outbreak activity and interesting findings from all regions of the global network. It also allows program management staff to communicate outward of notable changes in trends in specific regions or areas of concern.

## Conclusions

The Armed Forces Health Surveillance Center’s Division of Global Emerging Infections Surveillance and Response network continues to expand internationally to effectively identify and respond to threats from a wide range of disease agents and syndromes. The growth and enhancement of this surveillance system in anticipation of pandemic/avian influenza allowed the DoD to identify the recent influenza pandemic [21,22] and a number of other infectious disease outbreaks in communities around the world. A global system with defined goals and pillars of focus, the AFHSC-GEIS network has evolved to become a model for emerging infectious surveillance platforms at the local, regional and international level. By utilizing this established global system and the IHR(2005) as a guide, the DoD is able to provide a common and systematic approach to disease surveillance, human capacity building and a framework for effective public health response. As emerging and re-emerging threats develop in areas where partners work with host country collaborators and global health institutions, the AFHSC-GEIS network stands ready to respond and support.

## Abbreviations

AFHSC: Armed Forces Health Surveillance Center; AFRIMS: Armed Forces Research Institute of Medical Sciences; CDC: Centers for Disease Control and Prevention; CDHAM: Center for Disaster and Humanitarian Assistance Medicine; DoD: Department of Defense; EID: Emerging infectious disease; ESSENCE: Electronic Syndromic Surveillance for Early Notification of Community-based Epidemics; EUCOM: European Command; GEIS: Global Emerging Infections Surveillance and Response System; IHR: International Health Regulations; JHU/APL: Johns Hopkins University Applied Physics Laboratory; LRMC: Landstuhl Regional Medical Center; NAMRU-2: Naval Medical Research Unit No. 2; NAMRU-3: Naval Medical Research Unit No. 3; NHRC: Naval Health Research Center; NIC: National Influenza Center; NMRCD: Naval Medical Research Center Detachment; PACOM: Pacific Command; PHCR: Public Health Command Region; PHEIC: Public health emergency of international concern; RTA: Royal Thai Army; SMS: Short Message Service; USAFSAM: United States Air Force School of Aerospace Medicine; USAID: United States Agency for International Development; USAMRU-K: United States Army Medical Research Unit – Kenya; USUHS: Uniformed Services University of the Health Sciences; WRAIR-DVD: Walter Reed Army Institute of Research’s Division of Viral Diseases; WARUN: Walter Reed AFRIMS Unit Nepal.

## Competing interests

To the best knowledge of the authors there are no competing interests.

## Authors’ contributions

MCJ: Manuscript development and drafting. DLB: Manuscript development and review.
